# Delivery of a Clinical Genomics Service

**DOI:** 10.3390/genes5041001

**Published:** 2014-11-06

**Authors:** William G. Newman, Graeme C. Black

**Affiliations:** 1 Manchester Centre for Genomic Medicine, University of Manchester, Manchester, M13 9WL, UK; 2 Manchester Centre for Genomic Medicine, Central Manchester University Hospitals, NHS Foundation Trust, Manchester, M13 9WL, UK

**Keywords:** next generation sequencing, rare disease, exome, panel testing

## Abstract

Over the past five years, next generation sequencing has revolutionised the discovery of genes responsible for rare inherited diseases previously resistant to traditional discovery techniques. This review considers how this new technology is being introduced into clinical practice to aid diagnosis and improve the clinical management of individuals and families affected by rare diseases where access to genetic testing was previously limited. We compare and contrast the different approaches that have been adopted including panel based tests, exome and genome sequencing. We provide insights from our own clinical practice demonstrating the challenges and benefits of this new technology.

## 1. Clinical Genetic Testing for Inherited Disorders

Rare diseases are individually rare but affect a large number of individuals—for example, it is estimated that collectively they affect around 1 in 17 individuals in Western populations [1]. The identification of a specific genetic variant, in a patient DNA sample, that is responsible for a rare inherited disease can establish or confirm a clinical diagnosis, inform screening programmes and the implementation of personalised approaches to medical management. The information also facilitates risk assessment for affected families and enables reproductive decision-making.

Molecular genetic testing for rare diseases has been managed by a small number of expert clinicians, Clinical geneticists, over the past three to four decades, but is now becoming relevant to more patients seen across all clinical specialties. However, for clinicians within such so-called “mainstream” specialties (*i.e.*, outside of clinical genetics) it is often difficult to know how to access genetic testing for their patients. Over the past thirty years, Medical Genetics laboratories have been providing mutation testing for a relatively small number of inherited disorders due to variants in single genes. Of the approximately 7000 rare inherited disorders that have been defined, 3500 have so far been characterized at a molecular level [2]. The Genetic Testing Registry has collated the details on 16,000 tests for 4200 conditions analysing 2800 different genes [3]. The majority of these tests are still undertaken on a research basis. Clinically accredited testing provided by diagnostic laboratories is often limited.

**Figure 1:**
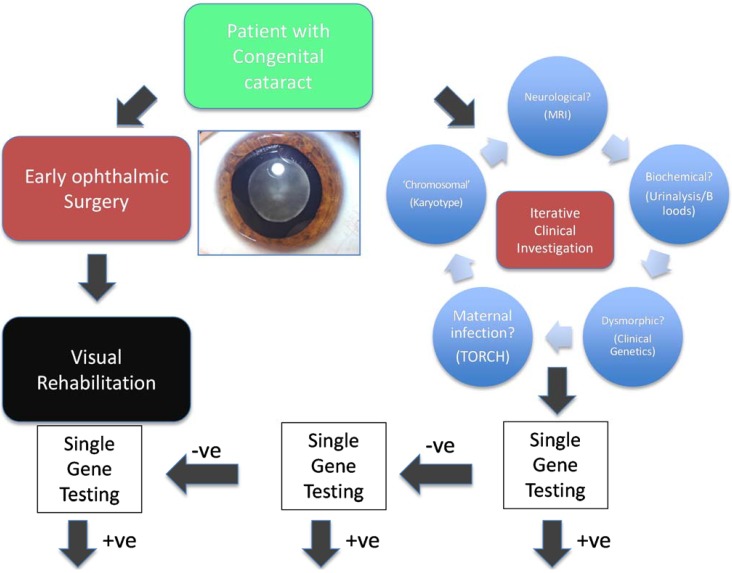
Classical clinical hypothesis-driven diagnostic approach. Traditional investigation of genetically and clinically heterogeneous conditions, such as congenital cataract, require an inefficient and iterative process based upon the development and testing of multiple clinical hypotheses, which leads to testing of many genes in series.

The traditional testing model has been driven by clinical hypotheses (Figure 1, using congenital cataract, a genetically heterogeneous condition, as an exemplar). A clinician usually defines, through detailed clinical investigation, a specific phenotype and subsequently develops a testable clinical hypothesis. The resulting clinical question leads to the request of a specific (usually single gene) test or at most the testing of a very small number of potentially relevant genes. This aims to confirm or refute the clinician’s suspicions and historically has been limited in great part by the technological limitations of nucleic acid sequencing. The pick up rate of such a testing approach varies considerably from approximately 0.6% for Fragile X syndrome [4] to over 40% for CHARGE syndrome [5]. In general, this has been a highly targeted approach, that is expensive, iterative and inefficient because of the limited number of target genes that can be tested and by the tendency to institute a large number of simultaneous investigations. By its very nature, it has also been limited to patients, and their relatives, with clinical features indicative of a specific genetic disease. Even where genetic testing is well established in familial breast cancer, genetic testing for *BRCA1* and *BRCA2* mutations has been limited to those with a very strong family history of the condition. Genetic molecular analysis has been especially challenging for genetically heterogeneous conditions, that is those conditions of identical phenotype cause by mutations in a wide range of genes, including intellectual and developmental delay, deafness, retinal dystrophies, congenital cataract, neuropathies and cerebellar ataxias.

## 2. Next Generation Sequencing as a Diagnostic Tool

In 2009, the first proof of principle studies were published exploring the application of massively paralleled or so called “next generation” sequencing (NGS) to identify the novel causes of rare inherited diseases [6,7]. These conditions had previously not been amenable to standard gene discovery approaches, e.g., *de novo* autosomal dominant disorders could not be refined by linkage analysis and/or candidate gene approaches had proved unsuccessful. The technology and bioinformatic approach demonstrated an extremely powerful ability to identify disease-causing genes from large genomic regions using small patient cohorts. This technology has led to the molecular characterization of numerous rare disorders and has been hailed as a revolution in medical research and practice [8]. NGS has already been applied in many disciplines across medicine, including in microbiology, virology, transplantation medicine and in the identification of acquired (somatic) mutations in tumours. However, this paper considers the use of clinical application of NGS in the molecular diagnosis of rare diseases.

NGS, when first applied to Mendelian disorders focussed on gene discovery where the majority of studies used either approaches focussing of targeted sequencing of genomic regions or most commonly on whole exome sequencing (WES). The WES approach is focused on approximately 1% of the genome, which includes coding and non-coding exons, some intronic and untranslated regions and promoters. It is important to note that the terms whole exome and whole genome sequencing are misnomers as the entire sequence of the exome or genome is not covered using the currently available techniques [9]. Focussing on the protein-coding DNA sequence such an approach generates manageable datasets; although large when compared to conventional sequencing, these are comparatively small when compared to the data from complete genomes. These present challenging, but surmountable, computing challenges [8].

**Figure 2:**
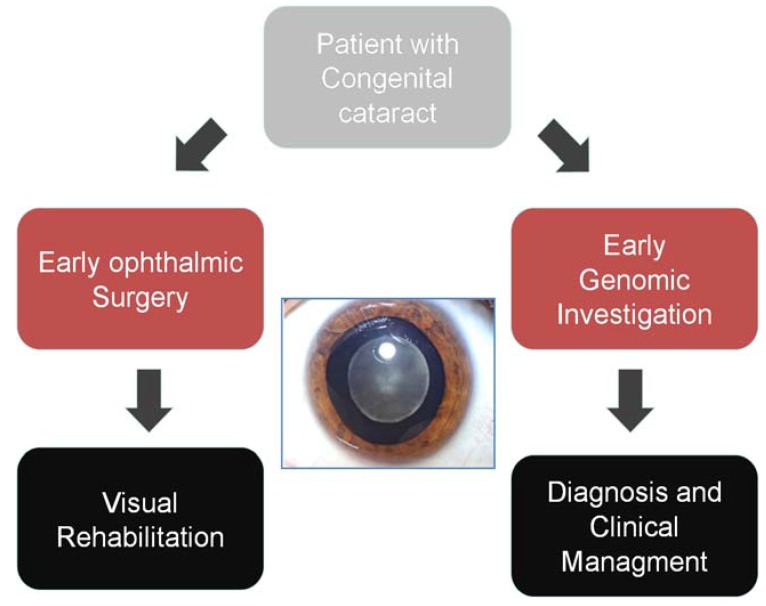
Genomic diagnostic approach Genomic technologies allow early genetic investigation of heterogeneous disorders, allowing much improved diagnostic pick-up, early diagnosis and reduced cost of investigation compared to a classical approach.

In the clinical setting, the commonest initial approach—that has been introduced by many clinical laboratories—is the targeted sequencing of a panel of genes relevant to a specific disease indication (Figure 2). Here, NGS has already had a major impact. Our own experience with testing of a panel of 105 inherited retinal dystrophy (IRD) genes has seen an increase in detection of the causal variant from 14% to 60% over the past two years of providing this service, allowing earlier implementation of genetic diagnosis and a reduction in the use of other diagnostic options [10]. More recently an “exome” approach to clinical diagnostic NGS sequencing has been adopted due to considerable practical advantages from the ability to develop a single diagnostic pathway for a huge range of clinical indications [11]. Please provide the original file (in ppt or other format) or a copy in tiff format of Figure 2 with high resolution.

### 2.1. Targeted Next Generation Sequencing for Diagnostic Molecular Testing

To sequence relatively small numbers of genes many approaches have been introduced to harness the power of NGS to improve throughput, reduce costs and improve turnaround times. For example, long range PCR has been used in our laboratory for *BRCA1* and *BRCA2* mutation analysis to generate large overlapping amplicons, which can then be sequenced. For panels of genes many technologies have been used to target the specific sequences, e.g., amplicon generation, Haloplex and hybridisation capture. Each of these methods has advantages and disadvantages in terms of labor intensity, cost and the specificity of the sequence generated. Further, each method has some limitations when identifying small insertion/deletion mutations has meant that, when using it *as a replacement for Sanger-based diagnosis,* care needs to be taken in using it as an equally effective diagnostic mechanism of excluding mutations in given genes [12].

Testing of panels of genes sequenced by NGS has been introduced with considerable success. The capture of selected sequences has been employed on both a research and diagnostic basis to study groups of genes—focused around biochemical pathways or those known to cause specific phenotypes, usually to analyse 20–200 genes in genetically heterogeneous disorders, such as IRD [10].

Such panels have many advantages over exome-based approaches: since they sequence fewer targets than genome-wide approaches they currently remain cheaper in absolute cost terms, although not when cost per base is used as the basis for evaluation. Panel-based approaches can now achieve even and very high levels of coverage of the targets selected, ≥99% coverage, allowing considerable clarity in diagnostic reporting. Importantly, many of the methodologies for capture-based exome sequencing, in particular at lower levels of overall coverage, have resulted in patchy coverage with a significant dropout of many, in particular GC-rich exons [13]. From a diagnostic viewpoint this dropout has been seen as challenging as it hinders the ability of the clinical scientist to deliver a report that provides a confident definition that a comprehensive screening of the selected genes has been undertaken and that, for exonic and flanking sequences, it is unlikely that a given individual carries a pathogenic variant. Such clarity is important as there are increasing numbers of clinicians, who are unfamiliar with complex genetic terminology and mechanisms, requesting genetic testing to inform their clinical practice.

Gene panel testing has other attractions when applied in the diagnostic sphere. The ability to define the genes that are screened lowers the likelihood that unexpected and potentially actionable findings may be encountered. However, it should be recognised that even amongst panel testing unexpected findings will nonetheless be found: for example, for two panels designed for ophthalmic disorders by our group [10,14], a wide range of conditions are covered by approximately 100–200 genes, many of broad pleiotropic effect [15]. Taking the example of retinal disease, it should be remembered that the ability to diagnose, in those with apparently isolated retinal dystrophy, syndromic conditions such as Senior-Loken and Bardet-Biedl syndromes can be—*for the patient*—unexpected and can result in altered management. When compared to single gene testing this then requires a more detailed approach to consent and counselling when implementing NGS testing.

When compared to exome (WES) or whole genome (WGS) sequencing, gene panel testing thus offers apparent simplicity and has consequently been employed to improve diagnosis of genetically heterogeneous monogenic diseases (e.g., retinitis pigmentosa, congenital deafness, cardiomyopathy). The relatively small numbers of potentially novel or pathogenic variants identified enable a detailed and focussed approach to variant interpretation that is more manageable for clinical scientists analysing and interpreting variants discovered through WES. However, even at this level of complexity, there are challenges presented in patient reporting. For example, in a series of 700 patients with IRD that has been evaluated for variation in 105 genes, in 40 (approximately 12%) of those for whom a molecular cause for their condition was found were shown to be heterozygous carriers of a pathogenic variant in another gene known to cause autosomal recessive IRD (Black; personal communication [16]). Here, the issue of disclosure is not straightforward and requires clear policy decisions by the diagnostic team delivering NGS [17]. Furthermore, it is essential that these policies are complementary to, and understood by, the clinicians consenting to testing. Since families with higher levels of consanguinity may not be identified to clinical scientists, it may be necessary to report all incidental carriers in such circumstances. Such an approach may differ from WES, where the numbers of heterozygous recessive variants present in each individual is high and the identification of carrier status relating to conditions not similar to the primary indication for testing is potentially more complex.

As gene panels are adopted for clinically and phenotypically heterogeneous disorders, it becomes possible for gene testing to be employed earlier in the diagnostic pathway (Figure 2). The breadth of variants that are identified—even in small gene panels—means that interpretation is highly context dependent and in our experience this has led to the development of dialogue between the clinical reporting scientist and the diagnostic clinician. The development of multidisciplinary reporting processes allows sharing of complex phenotypic, family, clinical and genomic data. For example, amongst the multi-systemic diseases that cause congenital cataract, such as cerebroteninous xanthomatosis, Stickler or Cockayne syndromes, genomic discoveries may uncover unexpected or overlooked clinical features that require re-evaluation in the clinic. In addition, genomic discoveries in conditions such as inborn errors of metabolism may define a range of secondary clinical investigations that support genomic findings and facilitate precise diagnosis [18]. In delivering NGS multi-gene panels, the identification of variants of uncertain significance is common. While, from a clinician’s viewpoint these cannot necessarily be acted upon, they represent a considerable workload for the team reporting genomic sequencing. At the current time the laboratory methodologies for NGS and the informatics tools to process the data have been honed substantially and have reached a point where this can be relatively easily automated. However, variant interpretation is both gene and phenotype specific. While there may well be certain guiding principles that are generally applicable, nonetheless this remains a labour intensive and complex aspect of NGS panel testing that must be factored into the costs of delivering testing in a healthcare setting. The diagnostic power of gene panel testing via NGS is remarkable and, alongside research developments, has allowed NGS very rapidly to contribute to clinical care. However, in planning the adoption of such processes, the hidden costs of testing are easily overlooked including the need for segregation studies, increased uptake of cascade testing and the need to evaluate the increasing demand for testing. These may be offset, potentially, by reduced adoption of clinical investigations that are superseded by NGS testing, but in many circumstances the costs of such tests are held in separate budgets to other aspects of clinical care. Finally, when considering multigene panels it is important to realise that, while compared to genome-wide approaches there is less data generated and analysed, there is nonetheless a considerable need for IT support, including sufficient computing hardware for data analysis and storage. Ensuring that data governance—in the diagnostic setting—fulfils those required in a healthcare setting immediately places a significant extra financial burden.

### 2.2. The Use of Genome-Wide NGS Approaches as a Diagnostic Tool

**Figure 3:**
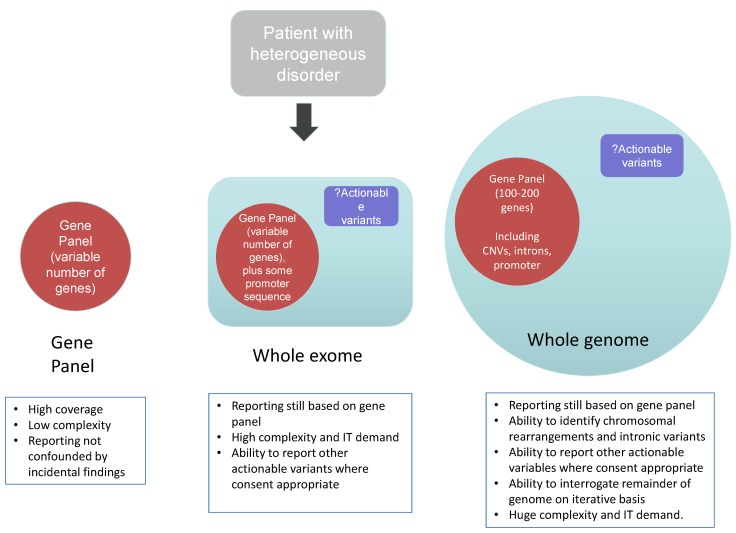
Diagnostic approaches using next generation sequencing.

The speed of technological advance in NGS is remarkable, and has led to the technology being described as “disruptive” [19]. Recapitalisation and standardisation of approaches that are key to secure delivery of accredited diagnostics remain challenging in an environment that is yet to fully mature. The panel-based approaches, discussed above, are inherently prone to redundancy as new genes relevant to a particular condition are discovered. As wet lab sequencing and bioinformatic processing and analysis become standardized and provided by increasing numbers of diagnostic laboratories, a single test and pipeline that leads to rapid diagnosis is appealing, with economies of scale and resultant rapid turnaround. Consequently genome-wide approaches, which facilitate sequencing of all known genes, are increasingly seen to be an important step in the delivery of genomic medicine—and we will now consider both exome-based and genome based approaches (Figure 3).

#### 2.2.1. Clinical Exome Sequencing

The ability of NGS to sequencing the entire exome—that is all of the coding exons of the expressed component of the genome, has fuelled gene discovery and accelerated the understanding of the pathogenesis of many monogenic diseases. As a result, *clinical exome sequencing* has been launched at a number of Centres in the United States [5], Australia and Europe and is being actively developed by clinical laboratories across the world [20]. Interestingly, in order to develop workable pipelines and a cost effective manner, at present in most clinical centres clinical reports are generated providing genetic sequencing data that is directly related to the specific phenotype of the tested individual—that is such an approach is based upon an *in silico* panel of genes that are analysed bioinformatically and reported (Figure 3). Such a targeted approach to analysis reduces substantially the cost of analysis, validation and variant interpretation. However, as discussed above, it is important in the consent process that patients and their families understand such focused analytical approaches.

In addition to a focussed approach, extended clinical reports may also be delivered that can provide information about:

(i) Carrier status for a range of recessive disorders to inform future reproductive risks.
(ii) Inherited disorders that are not predicted on the basis of family history or clinical presentation and for which treatment or preventive screening may be appropriate—so-called actionable variants. An example would be the detection of a variant in the low density lipoprotein receptor (*LDLR*) that would consistent with a diagnosis of familial hypercholesterolemia for which dietary intervention and statin treatment can reduce the risk of cardiovascular disease.
(iii) Pharmacogenetic data that may reduce the risk of adverse drug reactions, e.g., detection of variants in thiopurine methyl transferase that predict adverse response to thiopurines, e.g., azathiopurine.

There has been, and remains, extensive debate about the optimal approach to clinical exome sequencing, including uncertainty in defining the optimal population who should be tested and what information should be reported back to health care professionals and tested individuals. In one recent study of 250 cases referred for clinical exome sequencing, 80% of referrals were of children with neurological problems. In this group molecular diagnoses were confirmed in 62 (25%) with analysis confined to genes known to cause inherited disorders [5]. Of note, demonstrating the power of this approach, a significant number of the causative genes defined in this cohort had been discovered in the previous twelve months. The utility of exome testing has been explored in a number of other clinical settings, including improving diagnosis of children on intensive care units [21] or in children affected by likely recessive disorders when born to consanguineous parents [22].

Overall, exome sequencing lends itself to a high diagnostic yield in a range of clinical scenarios, including the molecular diagnosis of heterogeneous disorders, including primary immunodeficiencies and metabolic disorders. This precise diagnosis will result in reduced expenditure on alternative diagnostic tests and importantly provide patients and parents of affected children with diagnostic certainty. In addition to providing diagnostic information, reports are emerging of exome sequencing that has led to successful changes in clinical management—for example in the diagnosis and treatment of early onset inflammatory bowel disease [23] and in sepiapterin reductase deficiency in twins leading to supplementation of L-dopa therapy with 5-hydroxytryptophan [24].

#### 2.2.2. Whole Genome Sequencing

Whole genome sequencing (WGS) is considered to be the most comprehensive form of genetic test currently available [25]. In contrast to exome sequencing relatively few studies have used (WGS) in rare disease gene discovery. Initially successes have mainly been confined to use of WGS in combination with other sequencing approaches [26] or to identify non-coding mutations that have an effect on genes known previously to cause the specific phenotype [27]. Combination approaches allow refinement of the data analysis from tens of gigabytes to megabyte levels. The control datasets for non-coding variants are less mature and the functional assays to determine the potential phenotypic effects of non-coding variants are challenging to undertake and interpret, such that confident identification of pathogeneic mutations in the non-coding genome for rare diseases remains a formidable challenge. However, WGS presents considerable technological advantages over exome sequencing in that, because it is not based around biased capture-based enrichment approaches, it generates data on an entire genome, often with a consistent average coverage. Consequently, coverage of GC rich regions is improved and there is a considerably improved ability to determine rearrangements and copy number variants. Most recently this has been applied to a cohort of 50 individuals with a diagnosis of severe learning disability (LD) [28], a series of conditions that are associated with extraordinary genetic heterogeneity that are frequently undiagnosed. The conditions can be associated with macroscopic and/or submicroscopic chromosomal rearrangements as well as *de novo* copy number variations (CNVs) and single-nucleotide variations (SNVs). These are currently diagnosed using combined microarray/NGS (targeted panel or exome) sequencing approaches and has been demonstrated that WGS represents a single genetic test that can characterize the full range of genetic variants and enable a clinician to reach a genetic diagnosis in the majority of patients with severe LD.

However, WGS is yet to be introduced widely into routine clinical practice due in large part to the technological and practical hurdles presented by the technology. The generation of terabytes of sequence data that require massive computing capacity to analyse means that WGS is mainly confined to large-scale research or commercial laboratories where it has been applied in disease gene discovery studies. Advances in computing will ensure that WGS will be introduced rapidly over the coming years to supercede both gene panel and WES.

#### 2.2.3. Methodological Considerations of Different NGS Approaches

In adopting genomic technologies—from panel-based testing to WGA—the standardisation and full *clinical* validation of downstream processing will be essential. Here, a challenge is in ensuring that clinicians and clinical scientists are fully aware of the capabilities, limitations and overall design of analysis pipelines. For example, for WES we currently use a library preparation that results in, an average read depth of 140× across the exome which results in 94% coverage of the reference exome at 30× depth and generates approximately 13 Gb of data. Consequently, there is somewhat uneven coverage across many genes, a limitation that is important to stress to clinicians who may need to understand why a negative test may be received. For many capture technologies—that are used for both panel-based NGS approaches and for WES—the ability to assess dosage is limited and means that CNV analysis remains very challenging, potentially requiring reflex dosage testing. This is likely to be a limitation that WGS overcomes.

Analyses of raw data include data generation, collection and processing, followed by application-specific clustering, parsing and visualisation. Here there has been a pragmatic need to adopt research-designed bioinformatic analyses, which are often performed “in-house” using custom freeware designed pipelines for variant calling. Standardisation and full clinical stress testing will be key to ensuring that testing is of high quality, is reliably adopted and also to enabling effective data sharing across different diagnostic centres and platforms.

**Figure 4:**
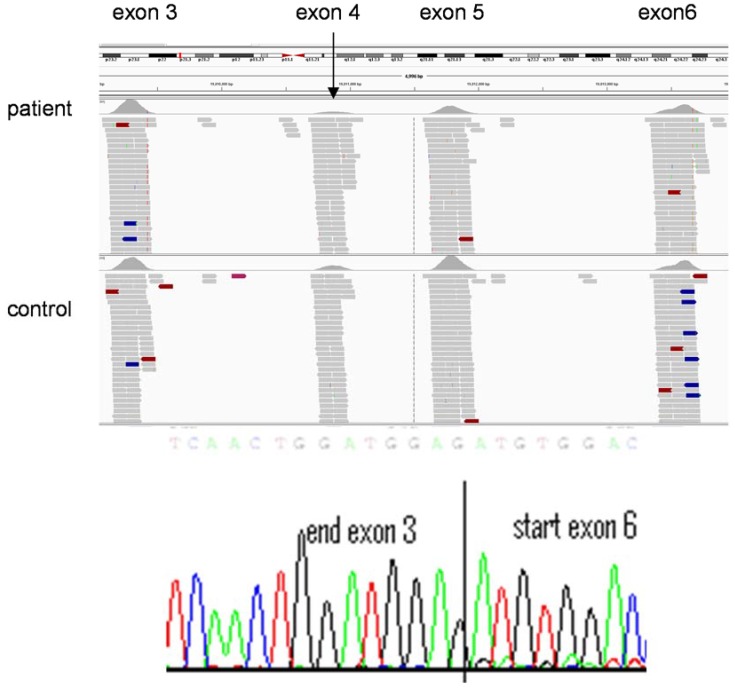
Copy number variation detected by exome sequencing. Decreased numbers of sequence reads are present in exons 4 (e.g., see arrow) and 5 of *LPL* in the individual with lipoprotein lipase deficiency (top panel) compare to exons 3 and 6 (similar number of reads in patient DNA sequence and that of a control individual below). This indicates a heterozygous deletion of exons 4 and 5 of *LPL*, which is confirmed in the bottom panel by sequencing of cDNA generated from RNA extracted from lymphocytes from the affected individual.

Variant interpretation remains extremely time-consuming and highly specialised. The process currently relies—in a diagnostic setting—on trained clinical scientists and has, to date, been far less automated than other areas of the NGS pipeline. *In silico* analyses determine whether sequence alterations are predicted to cause disruption of conserved residues. In diagnostic laboratories potential causal variants are often confirmed (currently, at least) by Sanger sequencing and segregation analyses, where possible, are undertaken to provide further evidence of pathogenicity. The definition of novel and pathogenic variants use sequence comparisons of sequences with (i) published data (themselves of highly variable reliability) (ii) databases of known mutations such as the Human Gene Mutation Database or publically available exome data resources such as Exome Variant Server [29] and (iii) the use of in-house databases of exome data. Such a labour intensive process remains important since a trained understanding of the technology and a high index of clinical suspicion can lead to re-evaluation of sequence data to define a causative mutation. For example, in a young child with severe triglyceridemia in whom a heterozygous, previously reported, mutation in *LPL* was identified. There was no sequence variant evident on the second allele to support a diagnosis of the autosomal recessive condition, lipoprotein lipase deficiency. However, the number of sequence reads was diminished across exons 4 and 5 of *LPL* (Figure 4) in comparison with an exome on the same sequence run. Subsequent, cDNA sequencing confirmed a heterozygous deletion of exons 4 and 5, confirming the diagnosis of lipoprotein lipase deficiency.

The limitations of current databasing are well understood amongst bioinformaticians and many clinical geneticists but will need to be more widely understood to enable secure variant interpretation across the clinical spectrum [30]. Of course, such resources are becoming more mature and informative as additional data is deposited and the ability to interpret exome-derived data is improving rapidly. By contrast, WGS will generate significant numbers of novel variants which will be difficult to interpret for pathogenicity and indeed it is likely that most early clinical analyses of WGS data will be focused on the *in silico* exome. For a thorough understanding of variant pathogenicity, high throughput functional studies including reporter assays, expression analyses, biochemical tests or *in vivo* assays will need to be developed to complement the emergent sequence data to allow full interpretation. Finally, the strategy around testing only the affected individual or in some scenarios WES or WGS of parents or other relatives (affected/unaffected) may be informative to refine the bioinformatics analysis and reduce the number of potential candidate causative gene variants. A successful example has been the application of a trio sequencing approach of affected child and unaffected parents to identify *de novo* pathogenic mutations, especially for severe congenital/developmental disorders [31].

## 3. Adoption of Clinical Genomics into Routine Clinical Practice

Next generation sequencing presents an exciting opportunity to revolutionise the diagnosis of rare disease and improve the effectiveness of healthcare delivery across all specialties. A number of specific areas will require focus if this is to be realised in a safe and effective manner:

### 3.1. Training

NGS is applicable across the healthcare spectrum—that is, it has been shown to be disease-agnostic. It is already proven to be a fundamental tool both clinical and research spheres and also—as recent studies have shown—relevant to both rare and common diseases. For example the next generation sequencing era introduces exciting new possibilities for singling out genetic variants of large effect that contribute to common disease in individuals as demonstrated for age-related macular degeneration [32]. A consequence of this broad relevance will be the opportunity to introduce NGS testing into the mainstream medical disciplines, including cardiology, neurology, and gastroenterology where to date genetic testing has been used less extensively and where the experience in delivering it remains more limited.

A recent survey of over 130 physicians at our Hospital across a number of specialties, including medicine (21%), surgery (13%), paediatrics (18%), anaesthetics (16%), and ophthalmology (7.5%) indicated enthusiasm for exome testing as a diagnostic aid. Over 11% of respondents had already requested an exome and over 53% envisaged requesting a test within the next five years. Limitations of current testing were availability (23%), difficulty with interpretation (47%) and concerns regarding identification of unexpected complex predictive data on cancer or neurodegenerative disease (23%). Such concerns emphasize the importance of clear guidance being established by national professional organisations in concert with patient support groups and other relevant stakeholders. However, experience from the practice of genetic medicine suggests that there is a need for an understanding of genetics, such as mutational mechanism and of genomic architecture and that this is aligned to experience in working closely with families and in delivering the counselling required to ensure effective and safe adoption of testing. Overall, therefore there is an urgent need for training to facilitate the adoption of the types of genomic technologies discussed above. This will need to be applied across all aspects of healthcare, including subspecialty clinicians and counsellors—potentially including those in primary care—who will need to be comfortable in understanding the nature and capability of the tests they order. Furthermore, this creates pressure to increase the numbers of scientists and bioinformatics experts who will be required to process the increasing number of tests.

The comparative youth of NGS is itself an inhibitor to widespread adoption in the clinical arena. In such a rapidly changing environment, the choices of technology and approach are fluid; exome capture technologies continue to improve, WGA costs are reducing and platforms rapidly maturing/becoming obsolete. Many healthcare-facing laboratories have until now been exercised with the decision to invest in the development of panel-based NGS tests or genome based (exome) approaches which are already considered by some to be out-dated. It is likely that the high cost of computing and of capitalisation/recapitalisation will either favour the larger healthcare organisations, or even lead to widespread outsourcing of sequencing. This is exemplified by the move by the 100000 Genome Project to a centralised and homogenised sequencing approach [33]. Both approaches will have a significant impact on how the technologies are introduced.

### 3.2. Standardised Phenotyping

The power of new genetic testing technologies to define the causes of rare inherited disorders has been remarkable. However, a limitation to further discovery has been the ability to share data generated on independent families with variants in the same gene with similar or different clinical phenotypes. Such data sharing will facilitate the definition of the ultra-rare conditions which, to date have remained undiagnosed [30]. Many research groups have identified potential causative genetic variants in single families where the burden of proof has not been satisfied to confirm causation as a mutation(s) in a second unrelated family has not been demonstrated. Many international efforts have been initiated to address this issue, including The Human Phenotype Ontology project [34] and databases that allow sharing of clinical and sequence data between clinical research groups, e.g., PhenomeCentral.

### 3.3. Ethical Issues

A range of complex ethical issues will influence a generalised introduction of genome-wide NGS.

At present, clinical reports from such genomic testing are generated to provide feedback relevant to the presentation of the tested individual. Thus, despite the breadth of genetic information available many centres, including our own, have decided initially to apply a bioinformatic filter based on the phenotypic features of the patient that predefines the panel of genes that will be analysed [21]. Such an approach significantly reduces, but does not abolish, the likelihood of identifying co-incidental genetic variants and speeds up the data analysis.

However, the potential to generate data that identify predisposition to conditions that are not predicted from family history or current health is significant. The American College of Medical Genetics [35] and European Society of Human Genetics [36] have considered how extra information potentially generated from genome analysis should be fed back to individuals. Information about increased risks of cardiac disease, cancer and rare inherited disorders (such as Marfan syndrome) potentially lend themselves to targeted interventions with improved outcomes. However, concerns have been raised about individual autonomy, inappropriate use of this information to discriminate in terms of employment and insurance and the burden placed upon health professionals to feedback accurate information that can have a measurable benefit [35,36,37].

A key area of future debate will be whether only those genes that are relevant to a specific patient phenotype are assessed and information relating to these fed back to the patient from their clinical exome—and if not, then precisely which so-called “actionable variants” are reported. The use of WES and WGS is a rapidly evolving area of medicine with different views emerging as to how this should be delivered. Our local patient advocate group has indicated that patients are keen for supplemental information that is derived from such testing to be used for patient advantage. However, the anecdotal feedback from patients interviewed in a clinic setting where exome testing has been offered, has suggested more reluctance in this regard.

Lastly, it is important to note the cautionary tales from newborn screening programmes. Tandem mass spectrometry has revolutionized the number of inborn errors of metabolism that can potentially be identified in the newborn period from blood spot analysis. However, results should only be fed back to parents where there is clear evidence of benefit for the newborn child through treatment or altered clinical management, or information that may influence future parental reproductive choices. The natural history of the metabolic disorder should be known, reference should be made to histidinemia and the inappropriate adoption of newborn screening when some children were exposed the risk of liver biopsy despite the condition having a benign natural history [38]. The results of any genome test should be societally and individually acceptable and understandable.

### 3.4. Economic and Societal Issues

The adoption of NGS—and ultimately WGS—will happen only if the diagnostic yield is sufficient to offset the costs of adoption. The 100000 Genome Project in the UK and similar initiatives across the world will start to address the technical and interpretative challenges posed by WGS and allow comparison with WES. However, it is challenging to measure the benefit of NGS as introduced across a population. Many groups, including our own, have numerous case reports of benefit through the identification of a previously unknown diagnosis. Clinical testing has already been introduced and so undertaking studies to establish improvements in outcome is difficult in this context. Randomized control trials will potentially provide the most compelling evidence of benefit and may be possible for defined groups of conditions, but it will be very challenging to interpret the benefits across heterogeneous groups of rare disorders. Such studies will be increasingly difficult to conduct if genome testing becomes the standard of care. Furthermore there are no universally agreed outcome measures in Genetic Medicine. Standard outcome measures such as the EQ-5D are not likely to capture the potential benefit of genetic testing, as they do not often result in an alteration in any of the measured parameters, e.g., mobility [39]. An alternative to randomized trials will be to make comparisons against historical data to determine potential benefit, but such analyses are beset by potential bias.

The point at which a genome test should be used in the diagnostic pathway is yet to be defined. Should a standard suite of diagnostic tests be used initially and sequencing applied as a second line or for certain clinical indications? Should the NGS test be the first line investigation? Studies to define these pathways are urgently required to ensure appropriate use of resources and to maximise patient benefit. At present genomic tests are used with rather limited scope within medical practice. This may reflect limited education of health care professionals about their utility, a lack of a robust evidence base for their routine adoption into clinical practice; and limited evidence that some genetic tests alter the clinical management.

## 4. Conclusions

NGS has already transformed the landscape for individuals and families with rare inherited disorders. Conditions previously resistant to research or accurate diagnosis are now the focus of study and amenable to routine diagnosis through panel based approaches or clinical exomes. The advances in genomic sequencing technology and computing will mean that such sophisticated tests will become the standard of care for individuals with rare inherited disorders. The obligation for geneticists and healthcare professionals to harness this genomic revolution for maximum patient benefit is a real one. The ethical, legal and social implications are complex and require an open vibrant dialogue and engagement from all members of society.
